# Supplementary material for: Eco-Friendly Solution Based on Rosmarinus officinalis Hydro-Alcoholic Extract to Prevent Biodeterioration of Cultural Heritage Objects and Buildings
Source: Int J Mol Sci. 2022 Sep 28;23(19):11463. doi: 10.3390/ijms231911463 (PMC9569761; doi:10.3390/ijms231911463)
Supplement: Supplementary file 1 [file ijms-23-11463-s001.zip › ijms-1911441-supplementary.pdf]

Supplementary material

Submitted to

Special Issue "New Types of Antibacterial Biocides"

Eco-friendly solution based on *Rosmarinus officinalis* hydro-alcoholic extract to prevent biodeterioration of cultural heritage objects and buildings

Viorica Maria Corbu†[1,2], Irina Gheorghe-Barbu†[2,3], Ioana Cristina Marinas*[2], Sorin Avramescu[4], Ionuț Pecete[5], Elisabeta Irina Geană[6], Mariana Carmen Chifiriuc[2,3,7,8]

[1]Department of Genetics, Faculty of Biology, University of Bucharest, Bucharest, Romania
[2]Research Institute of the University of Bucharest, University of Bucharest, Bucharest, Romania
[3]Department of Microbiology and Immunology, Faculty of Biology, University of Bucharest, Bucharest, Romania
[4]*Organic Chemistry Department, University of Bucharest, Bd. Regina Elisabeta 4-12, Sect. 3, Bucharest 030018, Romania*
5Central Reference Synevo-Medicover Laboratory, Bucharest, Romania
[6]National R&D Institute for Cryogenics and Isotopic Technologies – ICIT Rm. Valcea, Romania
[7]Academy of Romanian Scientists, Bucharest, Romania
[8] The Romanian Academy, Calea Victoriei 25, District 1, 010071 Bucharest, Romania

*__Correspondence__: Ioana Cristina Marinas - ioana.cristina.marinas@gmail.com
†These authors have contributed equally to this work

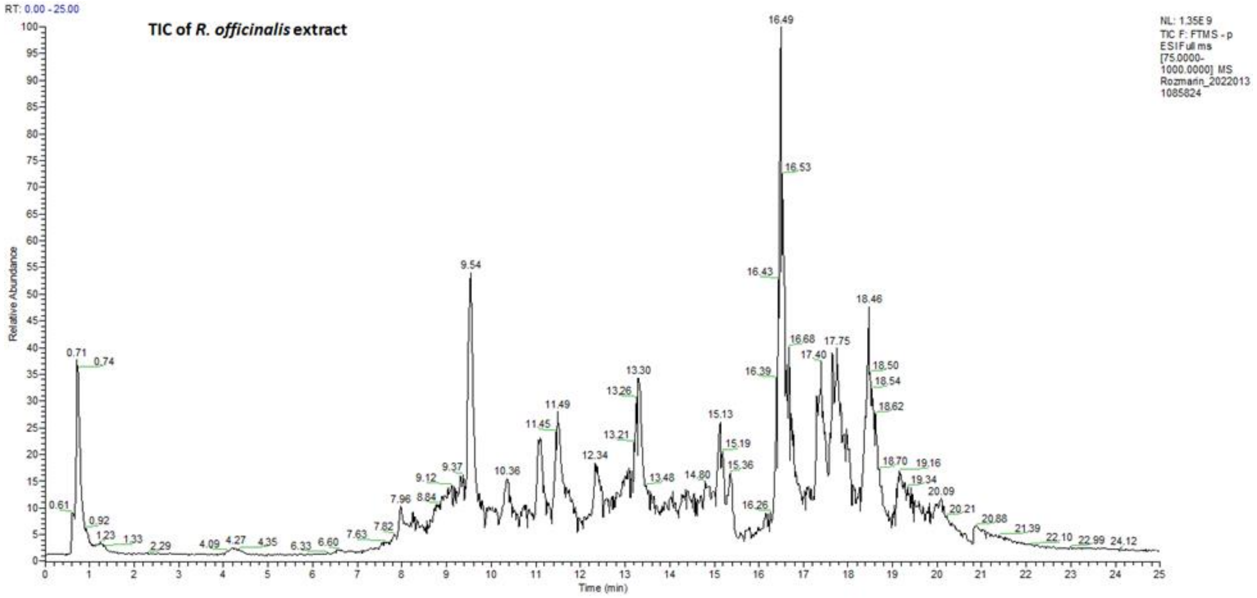

Figure S1. The total ion current (TIC) chromatogram *R. officinalis* extract by UHPLC–MS/MS detection in negative ionization mode

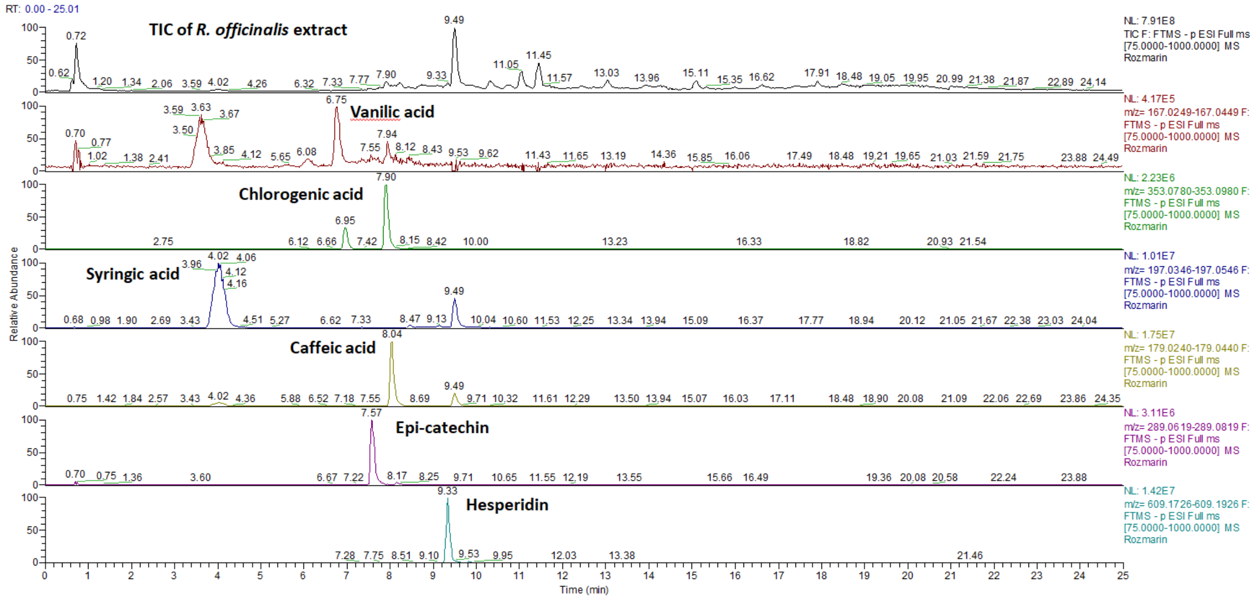

Figure S2. TIC and the extracted chromatograms of the main phenolic compounds quantified in *R. officinalis* extract (the chromatograms were extracted from TIC using a 5 ppm mass accuracy window; negative ion mode, full scan, base peak in the range 75-1000 m/z)

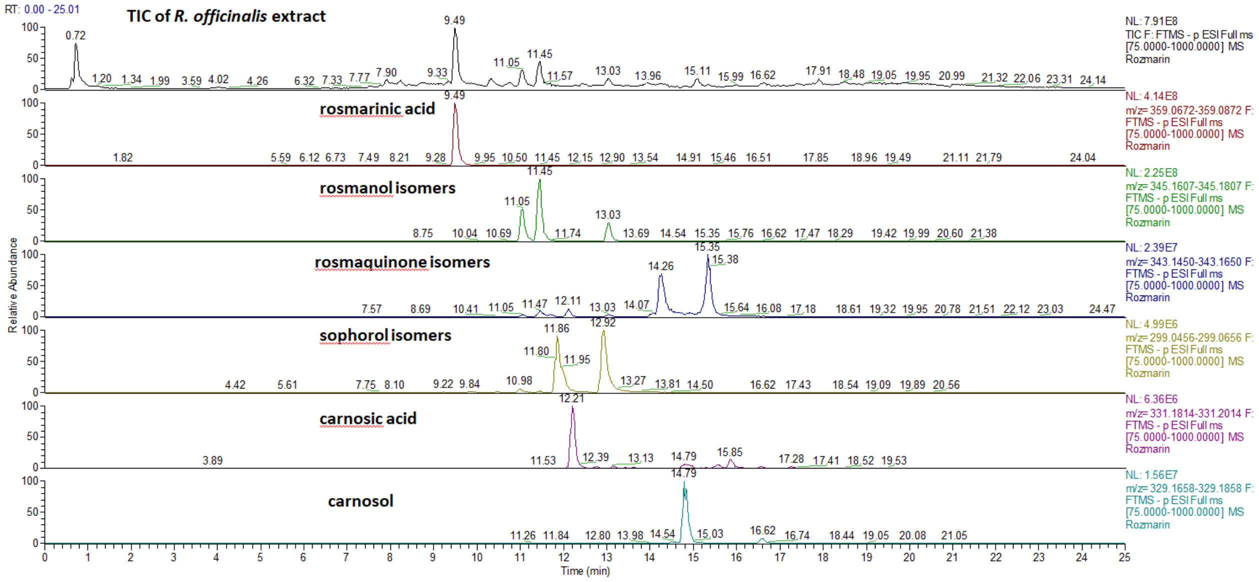

Figure S3. TIC and the extracted chromatograms of the main terpenoids in R. officinalis extract (the chromatograms were extracted from TIC using a 5 ppm mass accuracy window; negative ion mode, full scan, base peak in the range 75-1000 m/z)

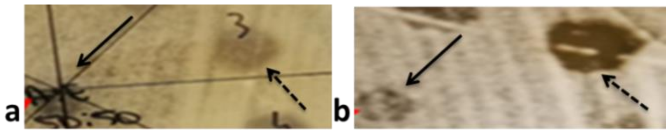

Figure S4. Example of the disk diffusion screening assay of the antimicrobial activity of the tested *R. officinalis* extract against *P. chrysogenum* strain isolated from the wooden churches (**a-** reverse; b-averse); full arrow- solvent inhibition zone; dashed arrow- *R. officinalis* extract inhibition zone.

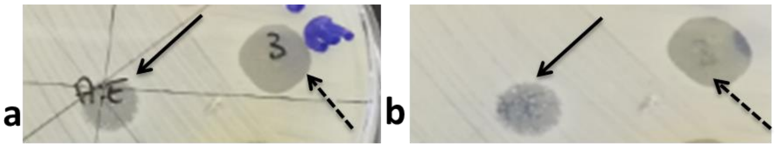

Figure S5. Example of the disk diffusion screening assay of the antimicrobial activity of the tested *R. officinalis* extract against *B. subtilis* strain isolated from the museum objects (**a-** reverse; b-averse); full arrow- solvent inhibition zone; dashed arrow- *R. officinalis* extract inhibition zone.

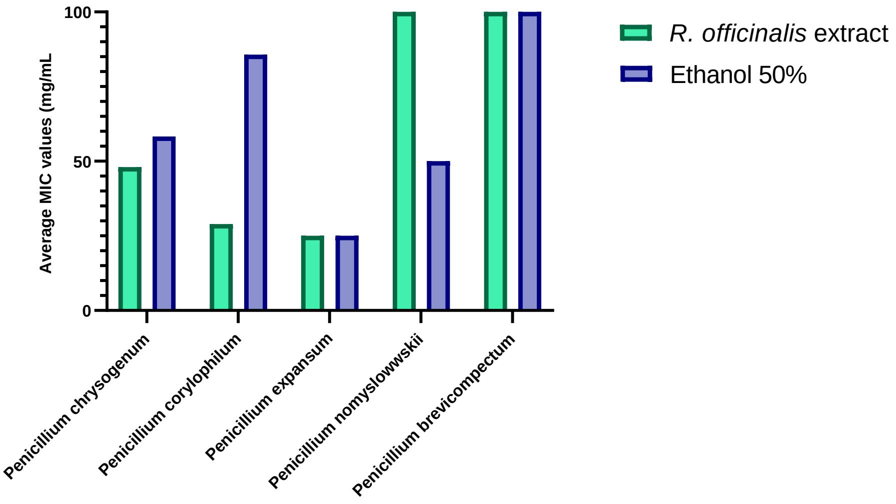

Figure S6 a. Graphic representation of the average values of the minimal inhibitory concentration (MIC) of *R. officinalis* hydro-alcoholic extract against *Penicillium* species.

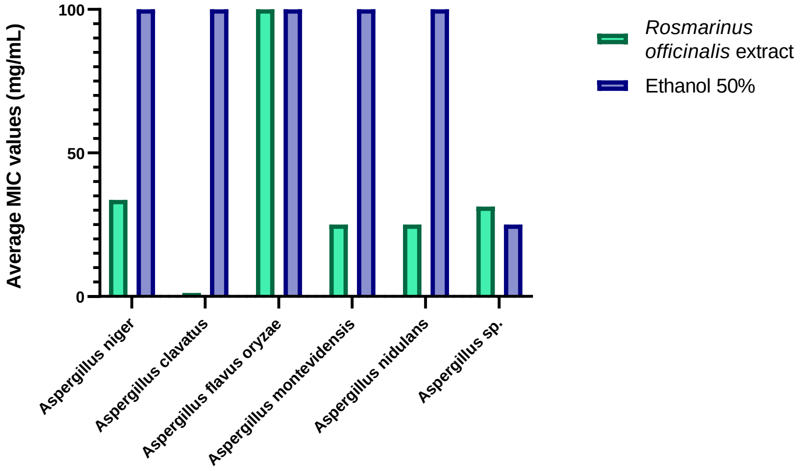

Figure S6 b. Graphic representation of the average values of the minimal inhibitory concentration (MIC) of *R. officinalis* hydro-alcoholic extract against *Aspergillus* species.

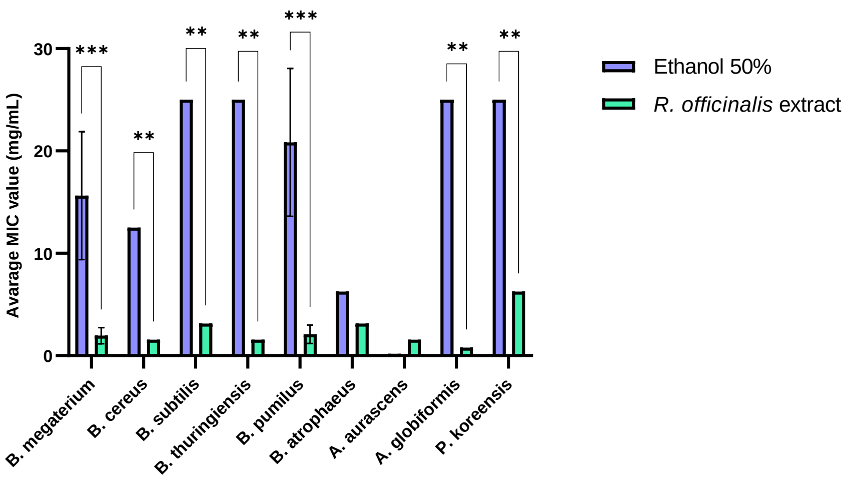

Figure S7. Graphic representation of the average values of the minimal inhibitory concentration (MIC) of *R. officinalis* hydro-alcoholic extract against bacterial strains by the species.

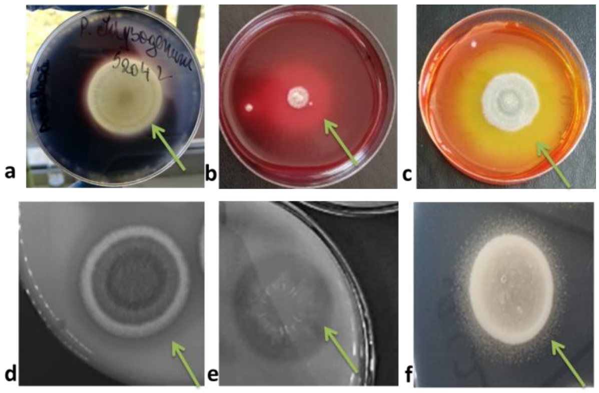

Figure S8. Examples of the influence of the *R. officinalis* extract and ethanol (50%) control on the ability of microbial strains to secrete compounds involved in the biodeterioration of heritage objects.
a) amylase- *P. chrysogenum*; b-cellulase-*P. chrysogenum*; c-organic acid-*P. digitatum*; d-caseinase- *P. chrysogenum*; e-caseinase- *B. cereus*; F- esterase- *B. thuringiensis.*
